# Supplementary figures and images for: Stochasticity in Ca2+ Increase in Spines Enables Robust and Sensitive Information Coding
Source: PLoS One. 2014 Jun 16;9(6):e99040. doi: 10.1371/journal.pone.0099040 (PMC4059641; doi:10.1371/journal.pone.0099040)

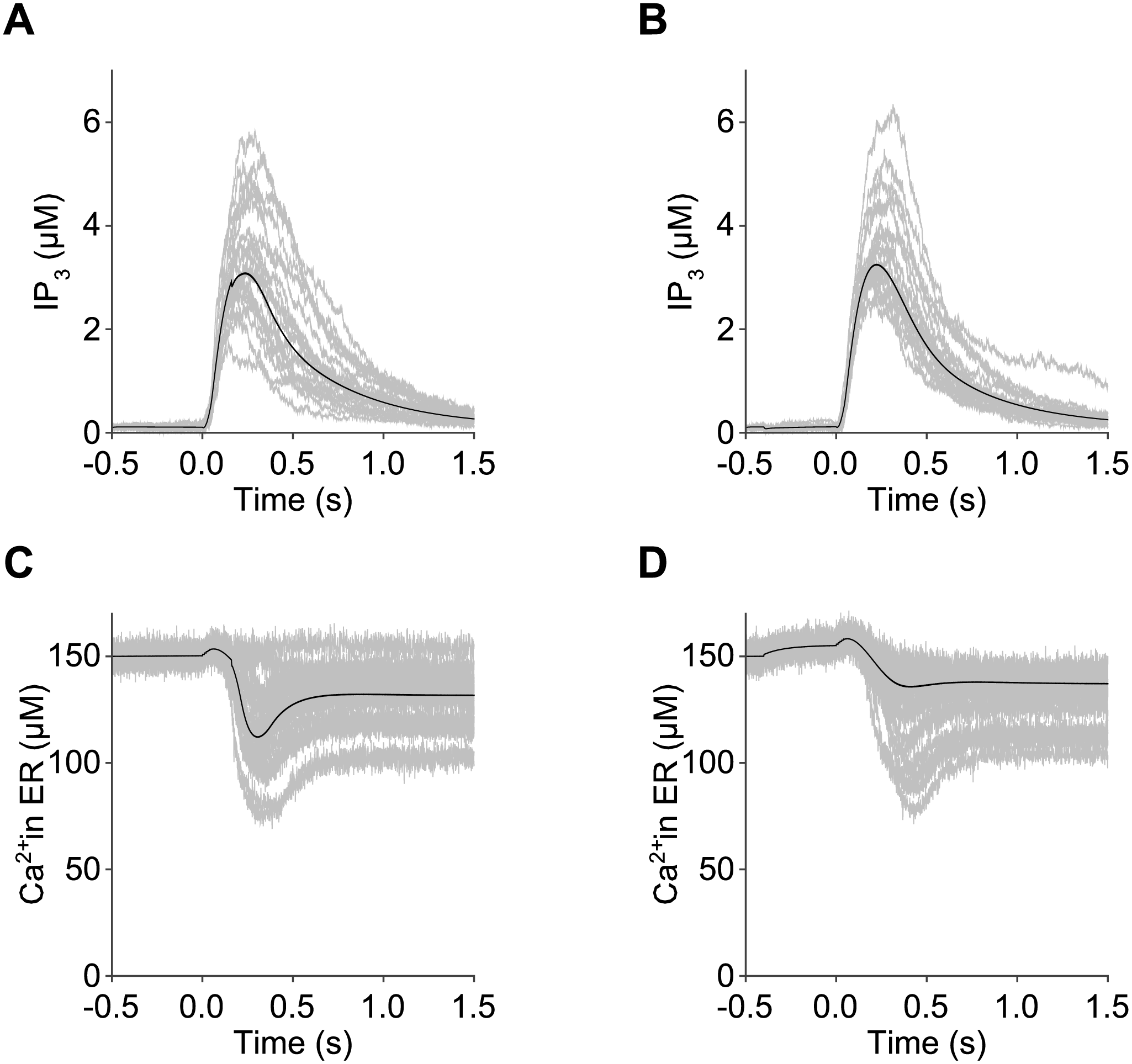

Supplement: Figure S1 — Time courses of concentrations of inositol trisphosphate (IP3) and Ca2+ in the endoplasmic reticulum (ER). A, B, Time courses of concentrations of IP3 in response to PF and CF inputs with Δt = 160 msec (A) and Δt = −400 msec (B) in the stochastic model in a spine volume (gray lines, n = 2,000 for each timing, 20 examples are shown) and in a cell volume (black lines, n = 20 for each timing), respectively. C, D, Time courses of concentrations of Ca2+ in the ER in response to PF and CF inputs with Δt = 160 msec (C) and Δt = −400 msec (D) in the stochastic model in a spine volume (gray lines, n = 2,000 for each timing, 20 examples are shown) and in a cell volume (black lines, n = 20 for each timing), respectively. (TIF) [file pone.0099040.s001.tif]

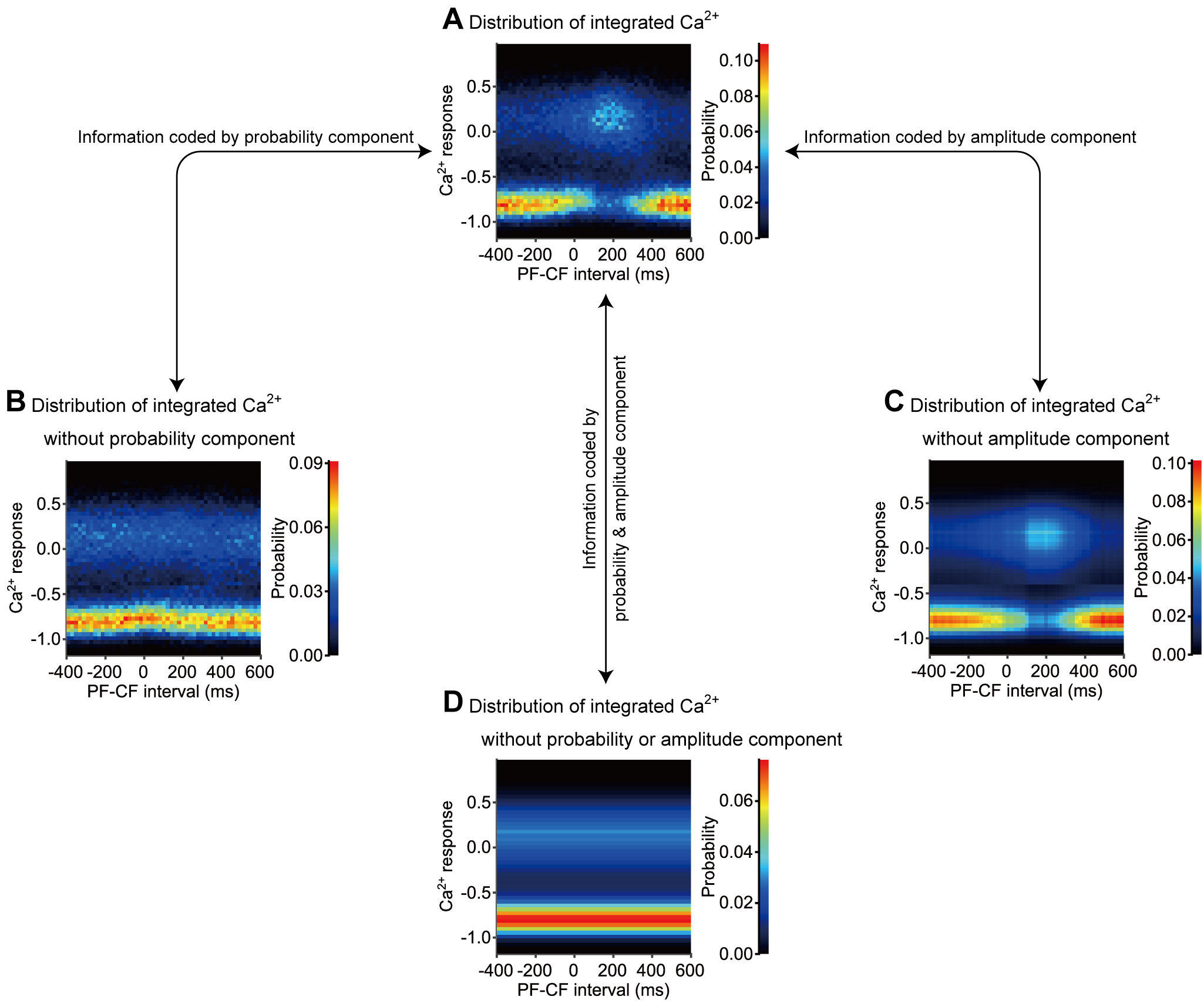

Supplement: Figure S2 — Distribution of Ca2+ response and that without the probability and/or amplitude components. A, Distribution of Ca2+ response. B, C, Distribution of Ca2+ response without probability (B) or amplitude (C) component. D, Distribution of Ca2+ response without both components. Input timing information coded by the probability and amplitude component was calculated by the Kullback–Leibler (KL) divergence of the distribution of B and C from that of A, respectively. Input timing information coded by the distribution of Ca2+ response was calculated by the mutual information between Ca2+ response and PF-CF interval, which is equal to the KL divergence of the distribution of D from that of A, and also equal to the sum of the input timing information coded by probability and amplitude component. (TIF) [file pone.0099040.s002.tif]

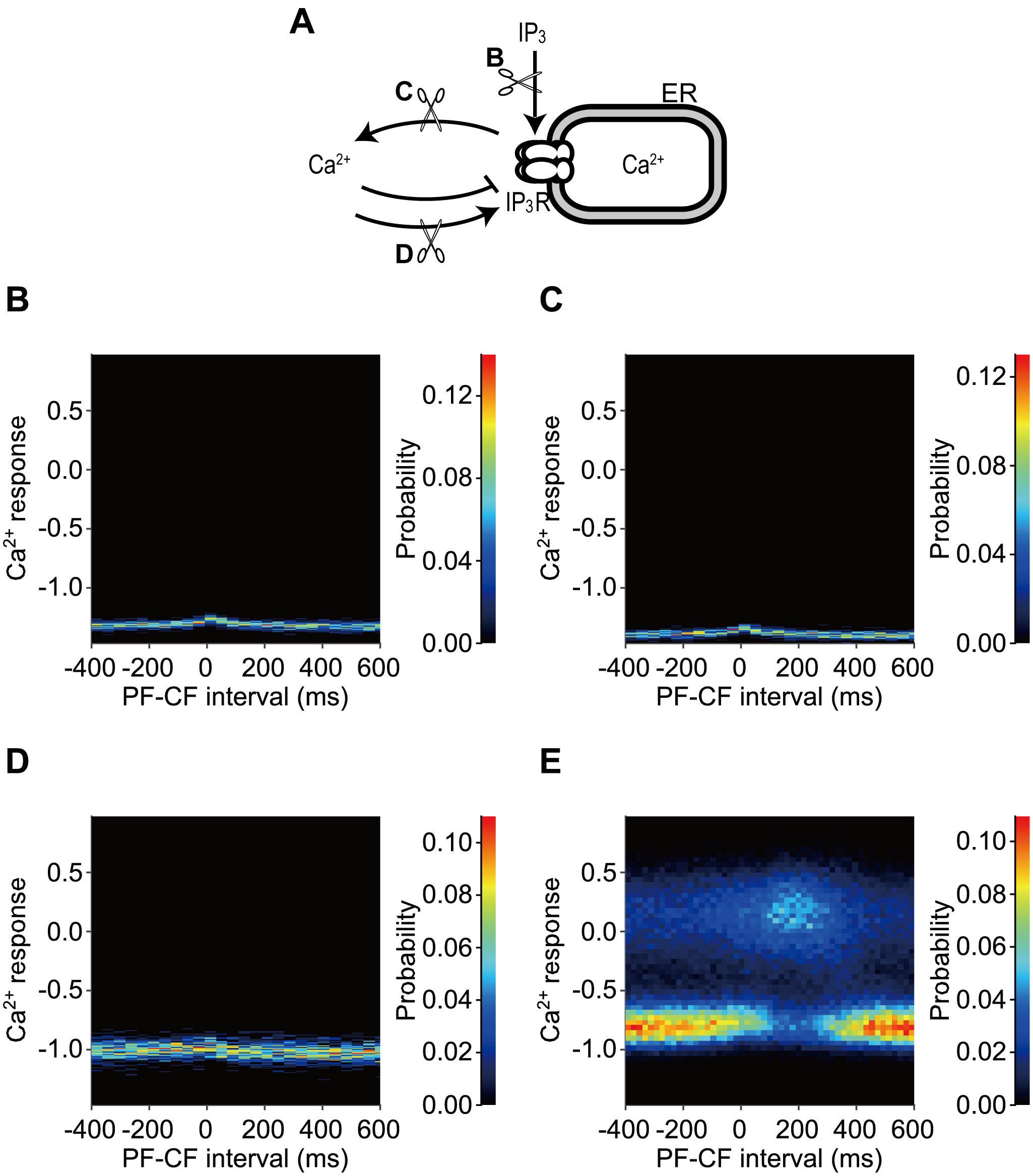

Supplement: Figure S3 — Mechanism of probability coding. A, Schematic representation of the pathway deleted in the following figures. B–D, Distribution of Ca2+ response along the PF-CF interval in a spine volume with blocking of the interaction of IP3 with the IP3 receptor (B), with blocking of the interaction of Ca2+ with the IP3 receptor (C), and with blocking of Ca2+ influx through the IP3 receptor (D) in the stochastic simulation. E, Distribution of Ca2+ response without deletion (control). (TIF) [file pone.0099040.s003.tif]

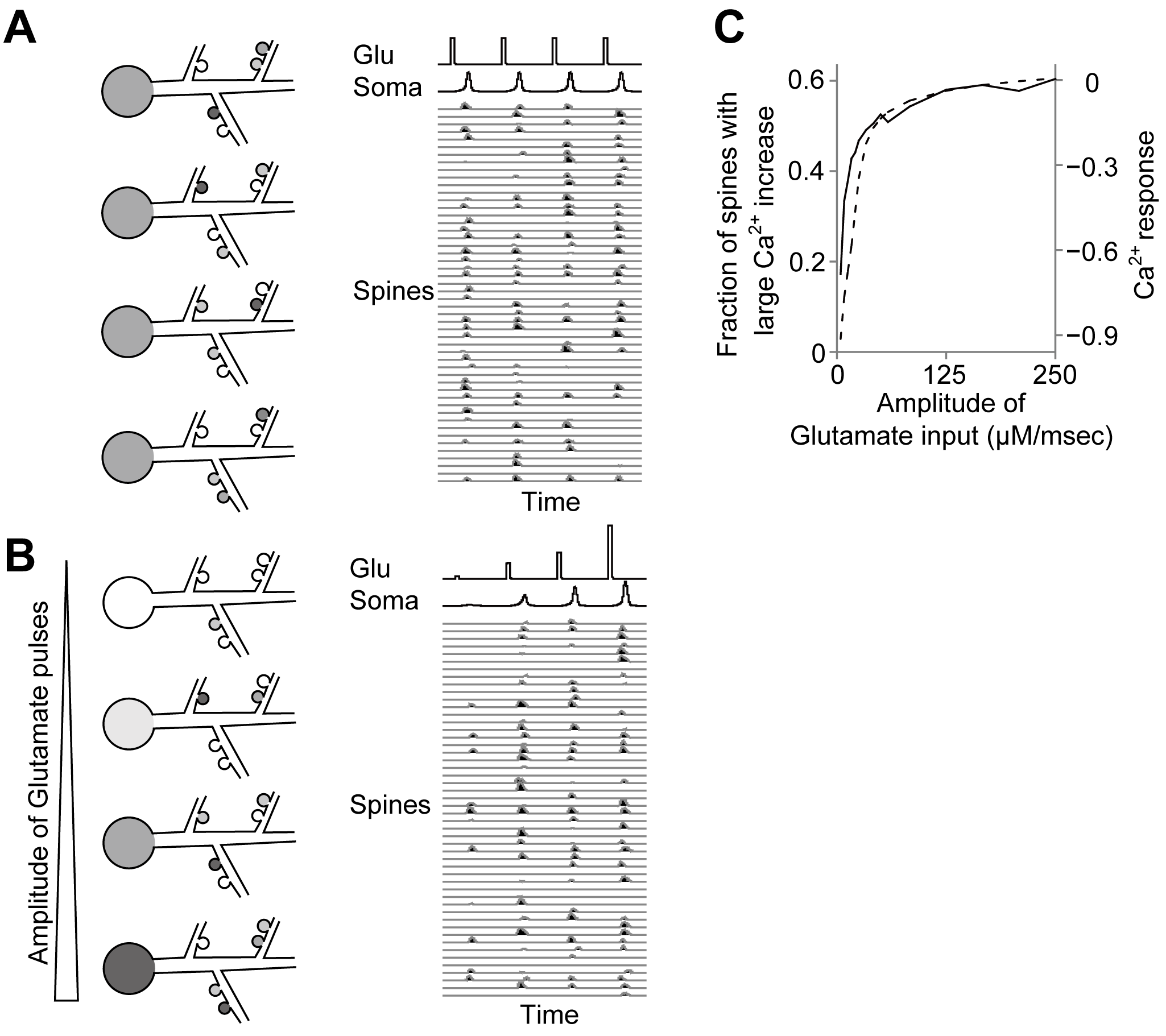

Supplement: Figure S4 — Possible experimental tests of probability coding in spines and amplitude coding in a soma. A, Ca2+ increase in response to repetitive addition of glutamate pulses with the same concentration in the stochastic model (spines) and in the deterministic model (soma). Colours code the concentration of Ca2+. Darker colours indicate higher concentrations of Ca2+. The time course of Ca2+ increase in the deterministic model and stochastic model are shown. B, Ca2+ increases in response to glutamate pulses of various concentrations in the stochastic model (spines) and the deterministic model (soma). Darker colours indicate higher concentrations of Ca2+. The time course of Ca2+ increase in the deterministic model and stochastic model are shown. C, Glutamate dose-responses curves of the fraction of spines with large Ca2+ increase above the threshold in the stochastic model (solid lines) and Ca2+ response in the deterministic model (dashed lines). Note glutamate inputs with amplitude CVs of 0.1 were used. (TIF) [file pone.0099040.s004.tif]

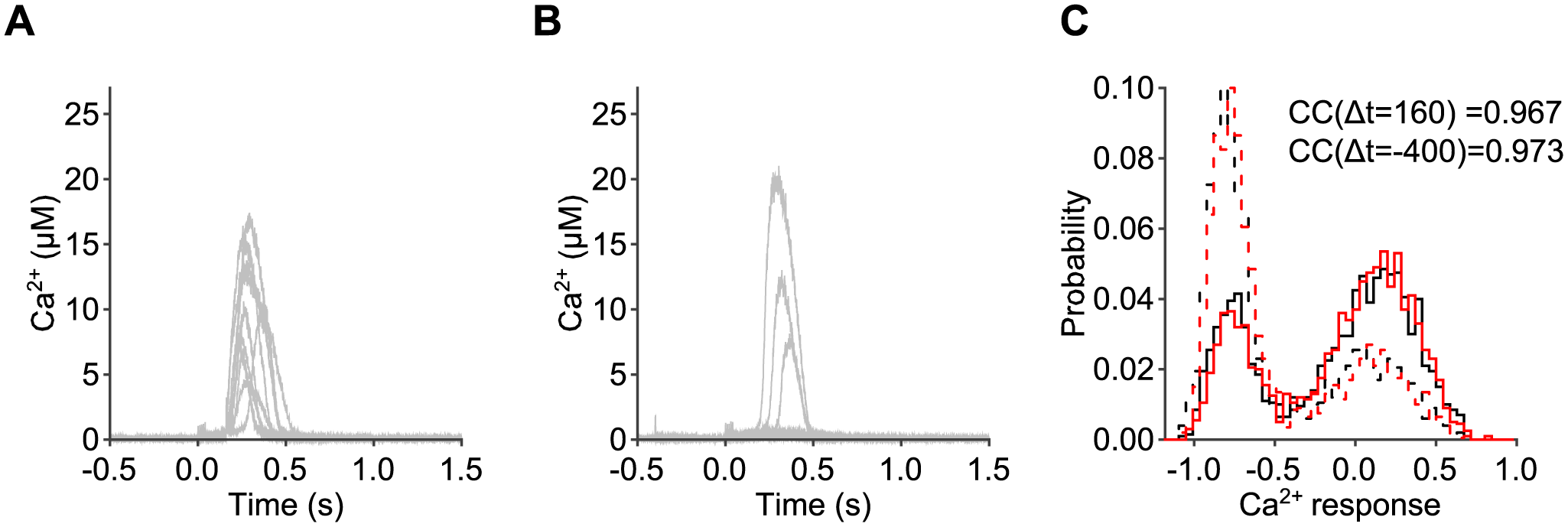

Supplement: Figure S5 — Validation of the numerical simulation. A, B, Ca2+ increase due to stimulation of PF and CF inputs with Δt = 160 msec (A) and Δt = −400 msec (B) by the stochastic simulation algorithm (SSA) (gray lines, n = 2,000 for each timing, 20 examples are shown). C, Distributions of the Ca2+ response in a spine volume with Δt = 160 msec (solid lines) and Δt = −400 msec (dashed line) by the modified tau-leaping method (black lines, same as Fig. 2C ) and SSA (red lines). The correlation coefficients (CCs) of the red and black lines were calculated. The large CCs demonstrates the validity of the modified tau-leaping method. (TIF) [file pone.0099040.s005.tif]

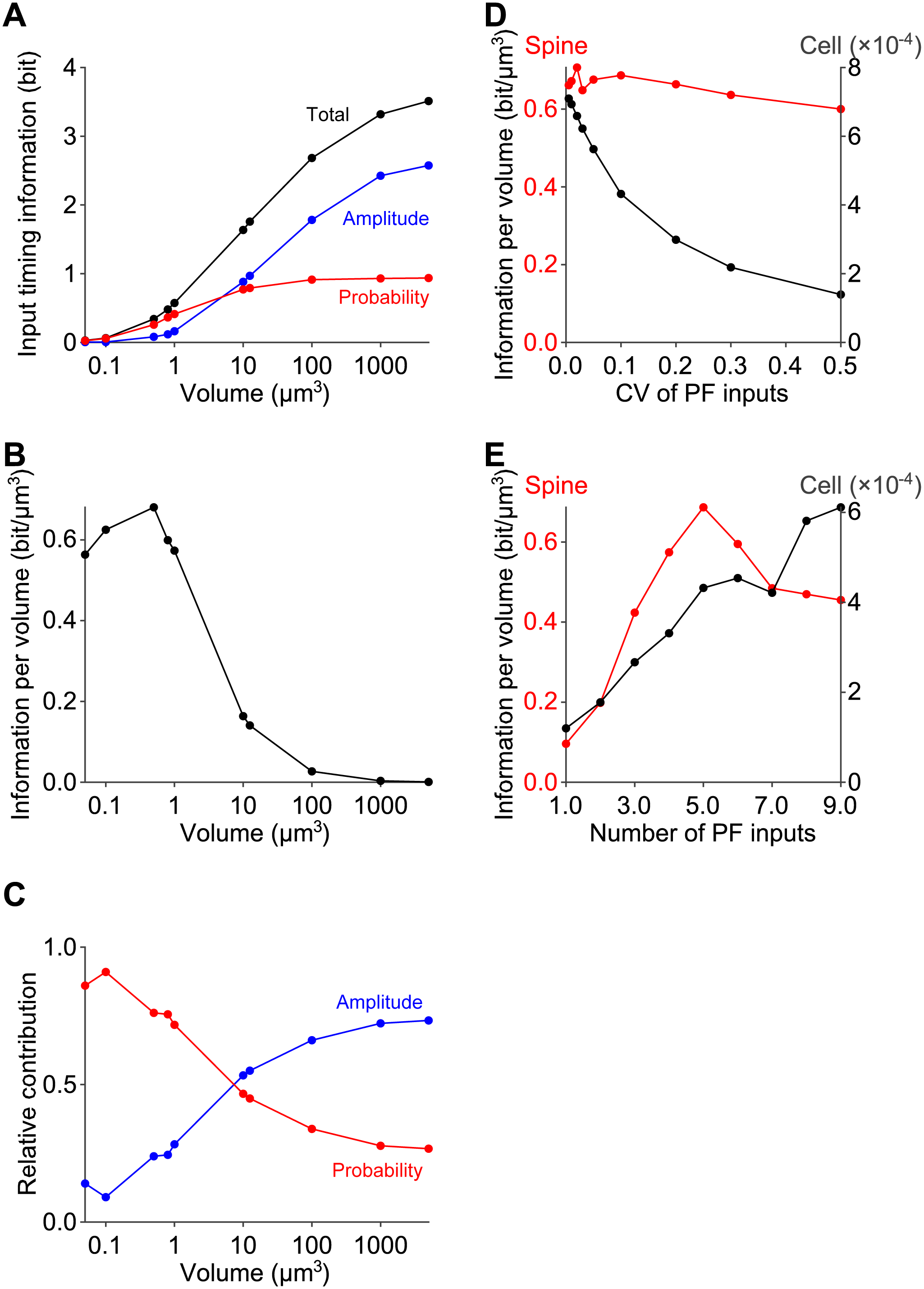

Supplement: Figure S6 — Input timing informations calculated in normal scale. Here we calculated the input timing informations in normal scale of Ca2+ response, and compared them with the input timing information calculated in logarithmic scale ( Fig. 3 and 4 ). Their similarity demonstrates that the results were qualitatively not affected whether they were calculated in logarithmic scale or normal scale. A, Volume-dependency of the input timing information coded by the total distribution of the Ca2+ response (black), by the probability component (red), and by the amplitude component (blue). B, Volume-dependency of the input timing information per volume. C, Relative contribution of the probability (red) and amplitude (blue) component to the input timing information. D, Input timing information per volume, coded by the Ca2+ response, in a spine (red) and in a cell (black). E, Input timing information per volume in a spine (red) and in a cell (black). Fig. S6A, B, and C correspond to Fig. 3D, E, and F , respectively. Fig. S6D and E correspond to Fig. 4B and D , respectively. (TIF) [file pone.0099040.s006.tif]

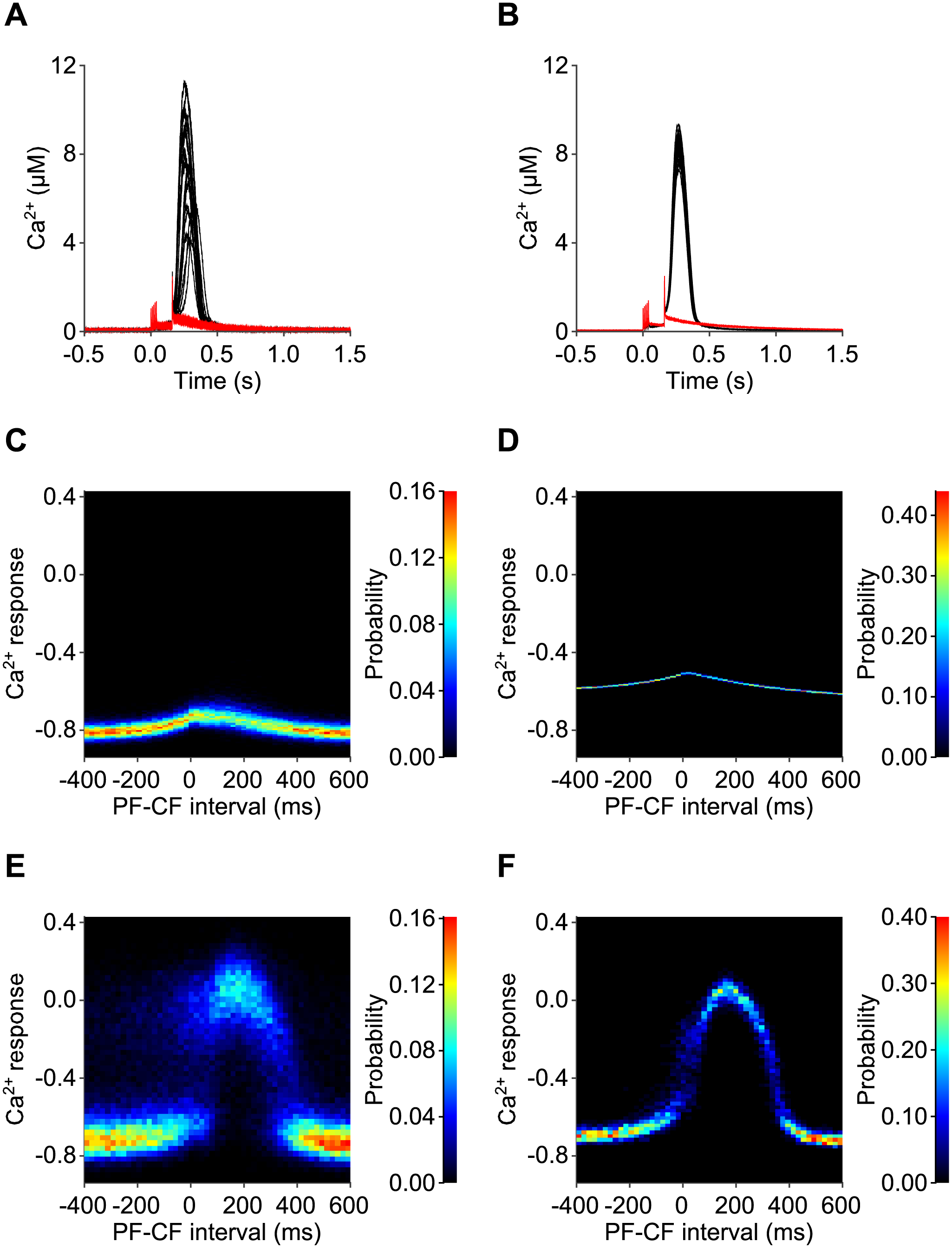

Supplement: Figure S7 — Results of simulation under the condition that the numbers of membrane molecules were proportional to the surface area. In this study, we assumed that the numbers of membrane molecules were proportional to the volume of the system. Here, to check the results of the simulation with the numbers of membrane molecules proportional to the surface area, we changed the model and performed the stochastic simulation. In this model, we altered the following three types of parameters: (i) initial numbers of the membrane molecules, (ii) membrane permeability coefficients, and (iii) constants for the propensity functions of the bimolecular and trimolecular reactions occurring on the membrane. (i) Initial numbers of the membrane molecules were set proportional to the surface area, thus they were multiplied by the surface-to-volume ratio (1/4 and 1/25 for the volume of 0.8 µm3 and 12.5 µm3, respectively). (ii) Membrane permeability coefficients were also set proportional to the surface area, and thus they were multiplied by the surface-to-volume ratio. (iii) Originally, in the tau-leaping method, constants for the propensity functions of bimolecular reactions are proportional to the inverse of the system volume. Thus, in this model, to set them proportional to the inverse of the surface area, constants for the propensity functions of the bimolecular reactions occurring on the membrane were divided by the surface-to-volume ratio. Likewise, the constants for the propensity functions of the trimolecular reactions were divided by the square of the surface-to-volume ratio. Surface molecules in this model are metabotropic glutamate receptor (mGluR), IP3 receptor (IP3R), plasma membrane Ca2+-ATPase (PMCA), sacro- and endoplasmic reticulum Ca2+-ATPase (SERCA), Na+/Ca2+ exchangers (NCX), and their complexes with other moleucles. Reactions occurring on the membrane were all reactions which involve the surface molecules. As a result, if the numbers of the membrane molecules are set proportio [file pone.0099040.s007.tif]

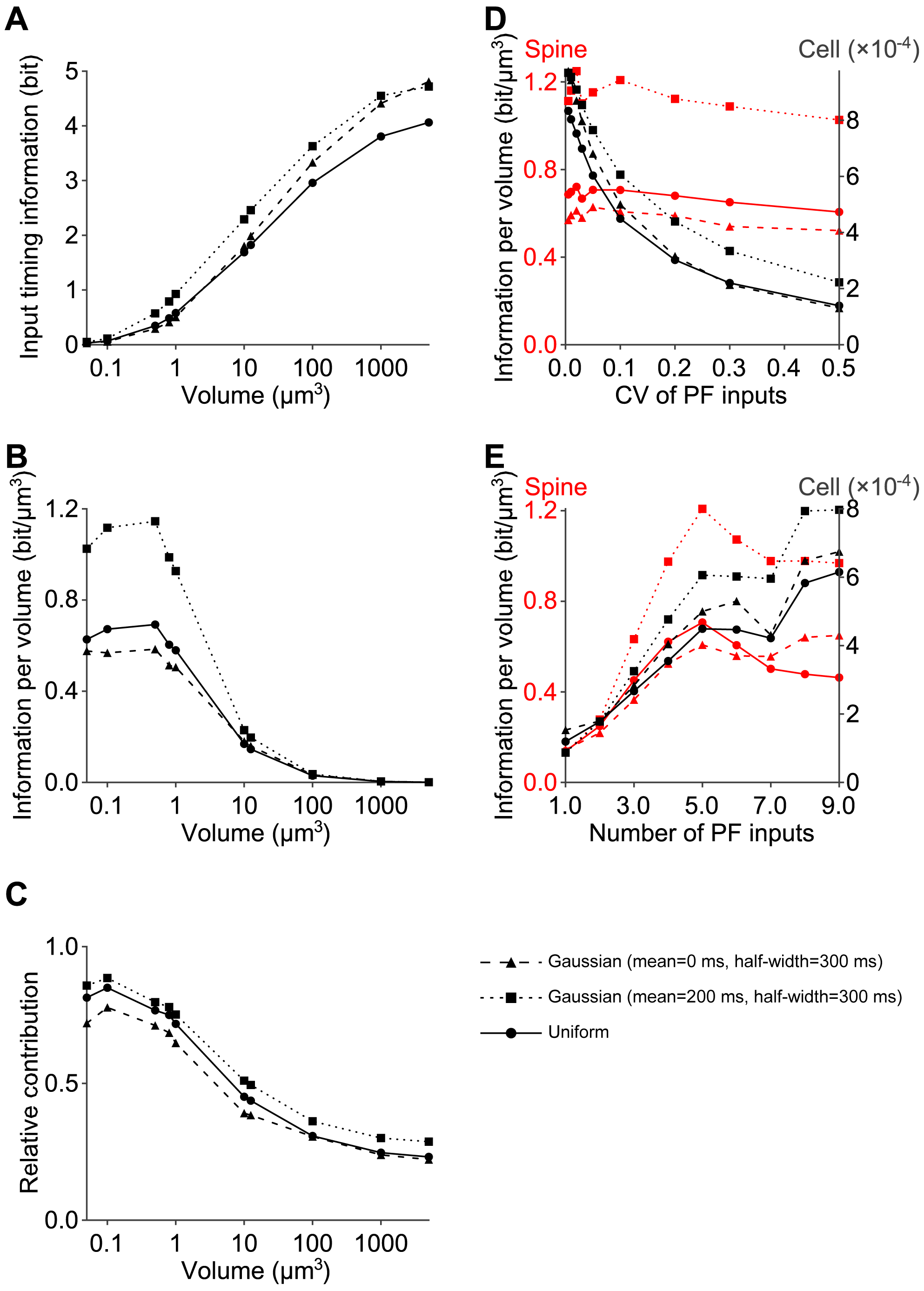

Supplement: Figure S8 — Input timing informations calculated on the assumption that the distribution of PF-CF interval follows Gaussian distribution. Here we calculated the input timing informations on the assumption that the distribution of PF-CF interval follows Gaussian distribution, and compared them with the input timing information calculated on the assumption that the distribution of PF-CF interval follows uniform distribution. Their similarity demonstrates that the results were qualitatively not affected by the distribution of PF-CF interval. PF-CF intervals were assumed to follow Gaussian distribution with mean of 0 ms and half-width of 300 ms (dashed lines with triangles), Gaussian distribution with mean of 200 ms and half-width of 300 ms (dotted lines with squares), and uniform distribution (solid lines with circles). A, Volume-dependency of the input timing information coded by the total distribution of the Ca2+ response. B, Volume-dependency of the input timing information per volume. C, Relative contribution of the probability component to the input timing information. D, Input timing information per volume, coded by the Ca2+ response, in a spine (red) and in a cell (black). E, Input timing information per volume in a spine (red) and in a cell (black). Fig. S8A, B, and C correspond to Fig. 3D, E, and F , respectively. Fig. S8D and E correspond to Fig. 4B and D , respectively. (TIF) [file pone.0099040.s008.tif]
