# Supplementary material for: Stochasticity in Ca2+ Increase in Spines Enables Robust and Sensitive Information Coding
Source: PLoS One. 2014 Jun 16;9(6):e99040. doi: 10.1371/journal.pone.0099040 (PMC4059641; doi:10.1371/journal.pone.0099040)
Supplement: Table S1 — Molecules and their initial number. This model consists of 56 molecules. Each of them is assumed to exist in one of the following compartment: cytosol, postsynaptic density (PSD), endoplasmic reticulum (ER), or extracellular space, with volumes of 0.1 µm3, 0.02 µm3, 0.002 µm3, and 10 µm3 in a spine respectively. The initial number of the molecules and the volumes of the compartments are the same as the previous deterministic model. (PDF) [file pone.0099040.s009.pdf]

Name	Initial number	Compartment	Description
Glu	0	PSD	Glutamate
mGluR	10	PSD	Metabotropic glutamate receptor type 1
Glu-mGluR	0	PSD	mGluRs activated by Glu binding
Gq-GDP	52	PSD	Trimeric G-protein Gq family
mGluR-Gq	8	PSD	mGluRs binding to Gq without Glu
Glu-mGluR-Gq	0	PSD	Intermediated state for Gq activation of Glu-mGluR-Gq complex
Ga-GTP	0	PSD	Activated Gqα subunit
Gbc	0	PSD	G-protein βγ comple
Ga-GDP	0	PSD	Inactivated Gqα subunit
PIP_2	5000	PSD	Phosphatidylinositol-4,5-bisphosphate
PLC-PIP_2	42	PSD	PLCβ subtype 4
PLC-Ca-PIP_2	8	PSD	PLCβ binding to Ca^{2+}
PLC-Gq-PIP_2	0	PSD	PLCβ binding to Gq
PLC-Ca-Gq-PIP_2	0	PSD	Fully activated form of PLCβ
PLC-Ca	1	PSD	The intermediate states of PLCβ that do not bind PIP_2
PLC-Ca-Gq	0	PSD	The intermediate states of PLCβ that do not bind PIP_2
DAG	0	PSD	Diacylglycerol
IP_3 (PSD)	0	PSD	Inositol 1,4,5-trisphosphate (IP3) in the postsynaptic density (PSD)
IP_3 (cytosol)	6	cytosol	IP_3 in the cytosol
IP3K	52	cytosol	IP_3 3-kinase
IP3K-2Ca	2	cytosol	Ca^{2+}-bound state of IP3K
IP3K-2Ca-IP_3	0	cytosol	Ca^{2+}- and IP_3-bound state of IP3K
IP5P	59	cytosol	IP_3 5-phosphatase
IP5P-IP_3	2	cytosol	Intermediate binding state of IP_3 5-phosphatase and IP_3
IP3R	14	cytosol	IP_3 receptor type 1
IP3R-IP_3	0	cytosol	IP_3-bound state of IP_3R
IP3R-IP_3-Ca	0	cytosol	Open state of IP_3R
IP3R-Ca	1	cytosol	Inactivation state of IP_3R, bound to one Ca^{2+}
IP3R-2Ca	0	cytosol	Inactivation state of IP_3R, bound to two Ca^{2+}
IP3R-3Ca	0	cytosol	Inactivation state of IP_3R, bound to three Ca^{2+}
IP3R-4Ca	0	cytosol	Inactivation state of IP_3R, bound to four Ca^{2+}
Ca (PSD)	0	PSD	Ca^{2+} in the PSD

Ca (cytosol)	4	cytosol	Ca^{2+} in the cytosol
SERCA	148	cytosol	Sacro- and endoplasmic reticulum Ca^{2+}-ATPase
SERCA-2Ca	7	cytosol	Ca^{2+}-bound state of SERCA
PMCA	68	cytosol	Plasma membrane Ca^{2+}-ATPase
PMCA-Ca	40	cytosol	Ca^{2+}-bound state of PMCA
NCX	32	cytosol	Na^{+}/Ca^{2+} exchangers
NCX-2Ca	0	cytosol	Ca^{2+}-bound state of Na^{+}/Ca^{2+} exchangers
Ca (ER)	1800	ER	Ca^{2+} in the endoplasmic reticulum (ER)
Calreticulin	960000	ER	Calreticulin
Calreticulin-Ca	72000	ER	Ca^{2+}-bound state of calreticulin
Ca (external)	12000000	external	Extracellular Ca^{2+}
MgGreen	14940	cytosol	Magnesium Green 1
MgGreen-Ca	60	cytosol	Ca^{2+}-bound form of MgGreen
PV	1380	cytosol	Parvalbumin
PV-Ca	1620	cytosol	Ca^{2+}-bound state of parvalbumin
CB	5850	cytosol	Calbindin-D_{28k}
CB-Ca	150	cytosol	Ca^{2+}-bound state of calbindin-D_{28k}
LAB	5997	cytosol	Non-cooperative low-affinity buffers
LAB-Ca	3	cytosol	Ca^{2+}-bound state of low-affinity buffer 1
LAB2	6000	cytosol	Cooperative low-affinity buffers
LAB2-Ca	0	cytosol	Ca^{2+}-bound state of low-affinity buffer 2
IP_4	0	cytosol	Inositol tetrakisphosphate
IP_2	0	cytosol	Iinositol bisphosphate
Glu (decayed)	0	PSD	Decayed glutamate

Table S1 | Molecules and their initial number.
