# Supplementary material for: Stochasticity in Ca2+ Increase in Spines Enables Robust and Sensitive Information Coding
Source: PLoS One. 2014 Jun 16;9(6):e99040. doi: 10.1371/journal.pone.0099040 (PMC4059641; doi:10.1371/journal.pone.0099040)
Supplement: Table S2 — Reactions and their parameters. This model consists of 43 reactions, one decay (decay of glutamate), one channel permeation (Ca2+ permeation through IP3R), two diffusions (Ca2+ and IP3 diffusions between cytosol and PSD), and two membrane permeations (Ca2+ permeation through the membrane of the cell and that through the membrane of the ER). In the stochastic model, decay of glutamate, two diffusions, and two membrane permeations were implemented as unimolecular reactions. Ca2+ permeation through IP3R was implemented as bimolecular reaction between Ca2+ and IP3R. All parameters are the same as the previous deterministic model. (PDF) [file pone.0099040.s010.pdf]

| Reaction | | | Forward rate constant | Backward rate constant |
|---|---|---|---|---|
| mGluR + Glu | ⇄ | Glu-mGluR | 11.1 | 100 |
| mGluR-Gq + Glu | ⇄ | Glu-mGluR-Gq | 11.1 | 100 |
| mGluR + Gq-GDP | ⇄ | mGluR-Gq | 2 | 100 |
| Glu-mGluR + Gq-GDP | ⇄ | Glu-mGluR-Gq | 2 | 100 |
| Glu-mGluR-Gq | → | Glu-mGluR + Gbc + Ga-GTP | 116 | 0 |
| Gq-GDP | → | Gbc + Ga-GTP | 1.00E-04 | 0 |
| Ga-GTP | → | Ga-GDP | 0.02 | 0 |
| Gbc + Ga-GDP | → | Gq-GDP | 6 | 0 |
| PLC-PIP2 + Ca (PSD) | ⇄ | PLC-Ca-PIP2 | 300 | 100 |
| PLC-Gq-PIP2 + Ca (PSD) | ⇄ | PLC-Ca-Gq-PIP2 | 900 | 30 |
| PLC-PIP2 + Ga-GTP | ⇄ | PLC-Gq-PIP2 | 800 | 40 |
| PLC-Ca-PIP2 + Ga-GTP | ⇄ | PLC-Ca-Gq-PIP2 | 1200 | 6 |
| PLC-Ca + Ga-GTP | ⇄ | PLC-Ca-Gq | 1200 | 6 |
| PLC-Ca-PIP2 | → | PLC-Ca + DAG + IP3 (PSD) | 2 | 0 |
| PLC-Ca-Gq-PIP2 | → | PLC-Ca-Gq + DAG + IP3 (PSD) | 160 | 0 |
| PLC-Ca + PIP2 | ⇄ | PLC-Ca-PIP2 | 1 | 170 |
| PLC-Ca-Gq + PIP2 | ⇄ | PLC-Ca-Gq-PIP2 | 1 | 170 |
| PLC-Gq-PIP2 | → | PLC-PIP2 + Ga-GDP | 8 | 0 |
| PLC-Ca-Gq-PIP2 | → | PLC-Ca-PIP2 + Ga-GDP | 8 | 0 |
| PLC-Ca-Gq | → | PLC-Ca + Ga-GDP | 8 | 0 |
| IP3K + 2 Ca (cytosol) | ⇄ | IP3K-2Ca | 1111.1 | 100 |
| IP3K-2Ca + IP3 (cytosol) | ⇄ | IP3K-2Ca-IP3 | 100 | 80 |
| IP3K-2Ca-IP3 | → | IP4 + IP3K-2Ca | 20 | 0 |
| IP5P + IP3 (cytosol) | ⇄ | IP5P-IP3 | 9 | 72 |
| IP5P-IP3 | → | IP2 + IP5P | 18 | 0 |
| IP3R + IP3 (cytosol) | ⇄ | IP3R-IP3 | 1000 | 25800 |
| IP3R-IP3 + Ca (cytosol) | ⇄ | IP3R-IP3-Ca | 8000 | 2000 |
| IP3R + Ca (cytosol) | ⇄ | IP3R-Ca | 8.889 | 5 |
| IP3R-Ca + Ca (cytosol) | ⇄ | IP3R-2Ca | 20 | 10 |
| IP3R-2Ca + Ca (cytosol) | ⇄ | IP3R-3Ca | 40 | 15 |
| IP3R-3Ca + Ca (cytosol) | ⇄ | IP3R-4Ca | 60 | 20 |
| SERCA + 2 Ca (cytosol) | ⇄ | SERCA-2Ca | 17147 | 1000 |
| SERCA-2Ca | → | SERCA + 2 Ca (ER) | 250 | 0 |

| | | | | |
|---|---|---|---|---|
| PMCA + Ca (cytosol) | ⇄ | PMCA-Ca | 25000 | 2000 |
| PMCA-Ca | → | PMCA + Ca (external) | 500 | 0 |
| NCX + 2 Ca (cytosol) | ⇄ | NCX-2Ca | 93.827 | 4000 |
| NCX-2Ca | → | NCX + 2 Ca (external) | 1000 | 0 |
| Calreticulin + Ca (ER) | ⇄ | Calreticulin-Ca | 0.1 | 200 |
| MgGreen + Ca (cytosol) | ⇄ | MgGreen-Ca | 1000 | 19000 |
| PV + Ca (cytosol) | ⇄ | PV-Ca | 18.5 | 0.95 |
| CB + 2 Ca (cytosol) | ⇄ | CB-Ca | 87 | 11.275 |
| LAB + Ca (cytosol) | ⇄ | LAB-Ca | 10 | 1000 |
| LAB2 + 2 Ca (cytosol) | ⇄ | LAB2-Ca | 10 | 4000 |
| Glu | → | Glu (decayed) | 250 | 0 |
| Ca (ER) + IP3R-IP3-Ca | ⇄ | Ca (cytosol) + IP3R-IP3-Ca | 2250 | 450 |
| IP3 (PSD) | ⇄ | IP3 (cytosol) | 980.39 | 19.608 |
| Ca (cytosol) | ⇄ | Ca (PSD) | 19.608 | 980.39 |
| Ca (ER) | ⇄ | Ca (cytosol) | 1.25 | 0.25 |
| Ca (external) | ⇄ | Ca (cytosol) | 0.001667 | 0.166667 |

**Table S2 | Reactions and their parameters.**
